# Association between systemic immunity-inflammation index and glucose regulation in non-diabetic population: A population-based study from the NHANES (2005–2016)

**DOI:** 10.1371/journal.pone.0313488

**Published:** 2024-11-12

**Authors:** Wenxiang Qing, Yujie Qian

**Affiliations:** 1 Department of Anesthesiology, Third Xiangya Hospital, Central South University, Changsha, China; 2 Department of Pediatrics, Xiangya Hospital, Central South University, Changsha, China; University of Cambridge, UNITED KINGDOM OF GREAT BRITAIN AND NORTHERN IRELAND

## Abstract

**Background:**

To investigated the link between the systemic immunity-inflammation index (SII), a new inflammatory biomarker, and the risk of abnormal glucose regulation in non-diabetic population.

**Methods:**

Using data from the 2005–2016 National Health and Nutrition Examination Survey (NHANES), we conducted a cross-sectional study on non-diabetic adults with data on SII and glucose regulation markers. We analyzed the relationship between SII and indicators of glucose regulation, including fasting plasma glucose, fasting insulin, hemoglobin A1c, oral glucose tolerance test (OGTT), and states of abnormal glucose regulation like impaired glucose tolerance (IGT), insulin resistance, and prediabetes.

**Results:**

Adjusting for confounders, higher SII levels were significantly associated with a higher OGTT and a greater likelihood of IGT (OR = 2.673, 95% CI: 1.845, 3.873). In subgroup analysis, participants without hyperlipidemia in the highest SII quartile had a 240% higher odds of IGT compared to those in the lowest quartile (OR = 3.407, 95%CI: 1.995, 5.820), an association not observed in those with hyperlipidemia (*p* for interaction < 0.05).

**Conclusions:**

SII emerges as a useful biomarker for identifying IGT in non-diabetic individuals, specifically in those without hyperlipidemia.

## Introduction

Type 2 diabetes (T2D) is a major global health concern, with an estimated 693million people expected to be diagnosed by 2045 [1]. Prediabetes, a condition that precedes the development of T2D and falls between normal glucose levels and diabetes, is expected to affect over 470 million individuals by 2030, with up to 70% of them progressing to diabetes [2]. A simulation study indicates that by preventing new cases of diabetes at different ages can lead to significant savings in lifetime medical expenses. For example, preventing a new case at age 40 could potentially save $124 600 in medical costs, with similar savings of $91,200, $53,800, and $35 900 if cases are prevented at ages 50, 60, and 65, respectively [3]. These findings emphasize the importance of strategies for diabetes prevention, not only for reducing healthcare costs but also for improving long-term health outcomes.

Research shows that chronic low-grade inflammation occurs before the onset of diabetes [4, 5], and the inflammatory and immune biomarker profile influences the development and progression of T2D [6, 7]. Insulin resistance (IR), where more insulin than usual is needed to maintain normal blood glucose levels, often precedes T2D and significantly affects glucose regulation [8, 9]. Inflammatory responses play a crucial role in the pathogenesis and development of IR [10]. People with prediabetes often show increased inflammation and islet dysfunction [11]. The systemic immune-inflammation index (SII) is a newly established index calculated based on lymphocyte, neutrophil, and platelet counts, which has potential uses in determining disease risk and prognosis [12–15]. However, the relationship between SII and abnormal glucose regulation in non-diabetic populations remains unclear. Thus, our study aimed to assess the predictive value of SII for abnormal glucose regulation in non-diabetic individuals.

## Materials and methods

### Study design and population

In this study, we conducted a cross-sectional analysis of the National Health and Nutrition Examination Survey (NHANES) data from 2005 to 2016. The study protocol received approval from the Centers for Disease Control and Prevention National Center for Health Statistics Institutional Review Board, and all participants provided informed consent. Details about NHANES can be accessed online at http://www.cdc.gov/nchs/nhanes/index.htm.

**Fig 1:**
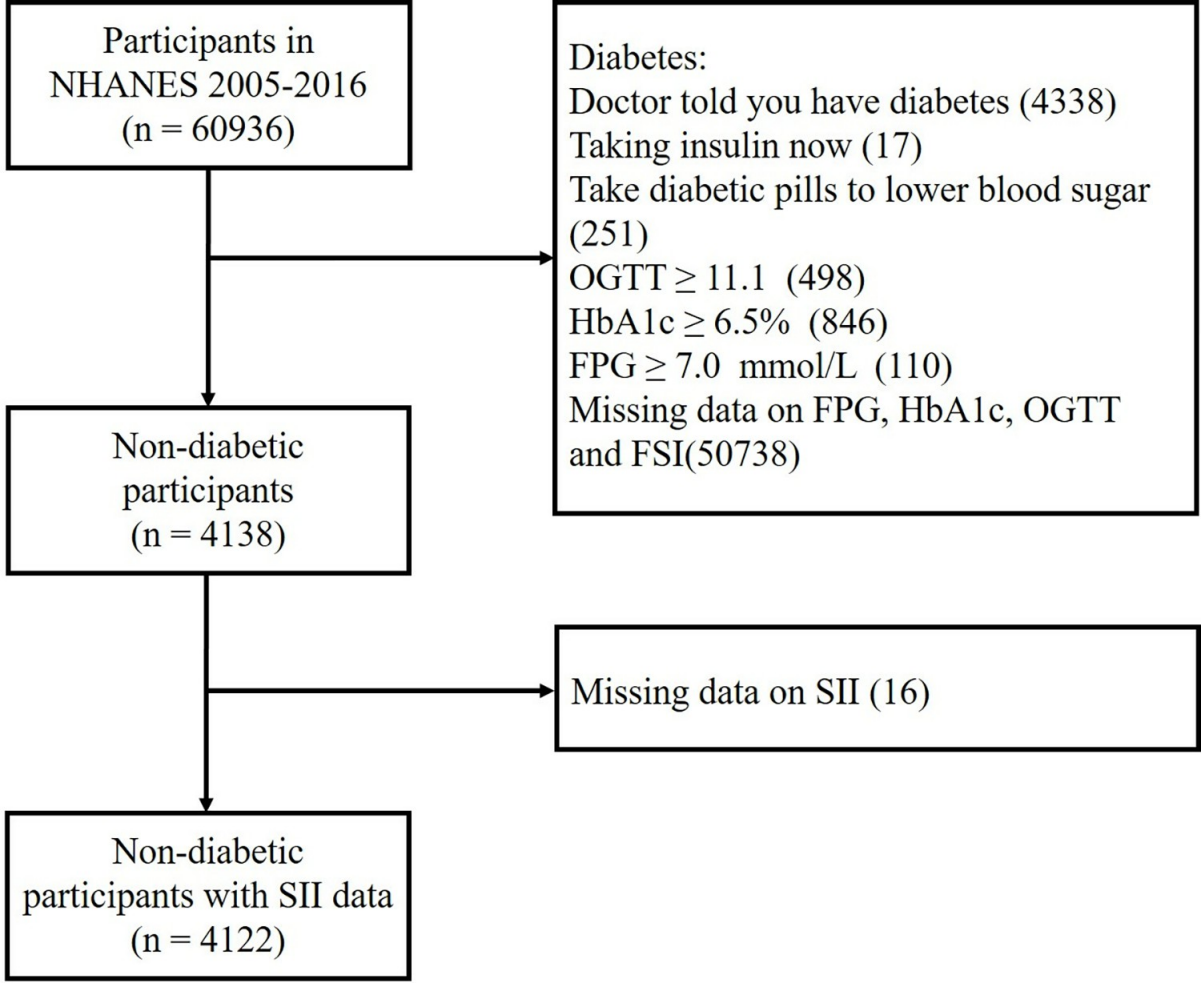
Flowchart of participant selection. NHANES, National Health and Nutrition Examination Survey.

A total of 60 936 individuals participated in NHANES from 2005 to 2016. Beginning in 2005, an oral glucose tolerance test (OGTT) was included in the lab protocol, conducted 2 hours (±15 minutes) after a 75-gram glucose load. The 2-hour blood glucose measurement was used to assess glucose tolerance in our analysis. The exclusion criteria were: a) participants with known diabetes (self-reported diabetes, or usage of insulin, or oral glucose-lowering medications); b) undiagnosed diabetes, determined by OGTT ≥ 11.1 mmol/L and/or hemoglobin A1c (HbA1c) ≥6.5%and/or fasting plasma glucose (PFG) ≥7.0 mmol/L [16]; c) participants with missing data on fasting plasma glucose FPG, HbA1c, OGTT, or fasting insulin (FSI); d) participants with missing data on SII. Ultimately, 4 122 participants were included in the study as depicted in Fig 1.

### Assessment of glucose regulation

Glucose regulation was assessed via FPG and OGTT using the hexokinase method and FSI using an immunoassay. HbA1c in whole blood was measured using the boronate affinity high-performance liquid chromatography system. The concentrations of FPG, FSI, and HbA1c were adjusted for differences in laboratory methodology in the NHANES 2005–2016.

Prediabetes was defined by HbA1c levels of 5.7% to 6.4% and/or impaired FPG (5.6–7.0 mmol/L) and/or impaired glucose tolerance (7.8–11.0 mmol/L) [17]. Insulin resistance (IR) status was assessed using the homeostatic model assessment of insulin resistance (HOMA-IR), with the 75th percentile value of HOMA-IR as the cutoff for defining IR in this study [18]. The corresponding cutoff value for the present study was 3.548. HOMA-IR scores were calculated using the formula: HOMA-IR = [fasting insulin (μU/ml) × fasting glucose (mmol/L)]/22.5 [19]. Diagnostic criteria for impaired glucose tolerance (IGT) included an FPG level <7.0 mmol/L and a 2-hour plasma glucose level ≥7.8 and <11.1 mmol/L [20]

### SII and covariates

SII, as an exposure variable, was calculated as platelet count × neutrophil count/lymphocyte count [14, 15]. Lymphocyte, neutrophil, and platelet counts were measured using automated hematology analyzing devices (Coulter®DxH 800 analyzer) and reported as ×10^3^ cells/ml.

Several covariates were considered in the analysis. Demographic characteristics, including age, race (Mexican American, non-Hispanic white, non-Hispanic black, other Hispanic, and other race), and gender, were taken into account. Lifestyle factors such as smoking status (current, former, never), drinking status (nondrinkers, low-to-moderate drinkers, and heavy drinkers), body mass index (BMI), and poverty income ratio (PIR) were included. PIR categorized individuals as either under poverty level (≤1) or above poverty level (>1) [21]. Laboratory testing variables, such as total cholesterol, high-density lipoprotein cholesterol (HDL-C), low-density lipoprotein cholesterol (LDL-C), and triglyceride levels, were also considered as covariates. Additionally, questionnaire responses related to self-reported physician diagnosis of coronary heart disease (CHD) and hypertension were used to ascertain the history of these conditions. Hyperlipidemia was defined as total cholesterol levels ≥200 mg/dL, triglyceride levels ≥150 mg/dL, LDL-C levels ≥130 mg/dL, or HDL-C levels ≤50 mg/dL for women and ≤40 mg/dL for men [22].

### Statistical analysis

SII was divided into quartiles from the lowest (Q1) to the highest (Q4). Continuous variables were reported as means with standard deviations (SDs), while categorical variables were presented as proportions. Differences between participants grouped by SII quartiles were assessed using a weighted t-test for continuous variables and a weighted chi-square test for categorical variables.

Multivariate logistic regression analysis was conducted in three models to examine the association between SII and glucose regulation, adjusting for covariates. Model 1 included no covariates, while model 2 adjusted for gender, age, race, and PIR. Model 3 further adjusted for additional covariates, such as BMI, drinking status, smoking status, self-reported physician diagnosis of CHD and hypertension, total cholesterol, triglyceride, and HDL-C and LDL-C levels.

Subgroup and interaction analyses were also performed to investigate the relationship in different subpopulations. The subgroups were stratified by gender (male/female), age (<40, 40–60, ≥60 years), BMI (<30, ≥30), hyperlipidemia (yes/no), drinking status (nondrinkers, low-to-moderate drinkers, heavy drinkers), and smoking status (current, former, never).

Data were uploaded to R Studio and analyzed with the use of the statistical packages R (version 4.3.1) and EmpowerStats (version 2.0). Statistical significance was set at *p*< 0.05. We utilized a weighting strategy to mitigate the potential volatility of our dataset.

## Results

### Participant characteristics

The characteristics of the study participants based on the quartiles of SII are presented in Table 1. A total of 4 122 non-diabetic participants were included in the study. Significant differences were noted among the SII quartiles in terms of age, gender, race, PIR, BMI, smoking status, hypertension, and serum lipid levels (total cholesterol, triglyceride, HDL-C, and LDL-C) (*p*< 0.05). Participants with higher SII scores tended to be older, female, non-Hispanic white, have a higher PIR, higher BMI, never smokers, have hypertension, and exhibited higher levels of total cholesterol, triglyceride, HDL-C, and LDL-C.

**Table 1:** Demographic characteristics of non-diabetic participants according to the quartiles of SII (National Health and Nutrition Examination Survey 2005–2016).

Characteristics	SII Quartiles				*p*-value
	Q1	Q2	Q3	Q4	
	N = 1031	N = 1030	N = 1030	N = 1031	
Age, years	35.281 ± 20.665	37.764 ± 20.357	38.694 ± 19.941	42.473 ± 20.888	<0.001
Gender (%)					<0.001
Male	59.1	54.4	51.6	42.3	
Female	40.9	45.6	48.4	57.7	
Race/ethnicity (%)					<0.001
Mexican American	18.3	22.3	24.8	22.1	
Other Hispanic	6.5	8	7.9	7.9	
Non-Hispanic White	34.8	46	45	51.1	
Non-Hispanic Black	35.7	19.1	18.4	15	
Other Race	4.7	4.6	3.9	3.9	
PIR (%)					0.002
under poverty level	23.2	16.9	22.4	22.3	
above poverty level	76.8	83.1	77.6	77.7	
BMI, kg/m^2^	25.616 ± 5.899	26.774 ± 6.087	27.683 ± 6.275	28.340 ± 7.728	<0.001
Drinking status (%)					0.902
Nondrinkers	13.3	15	13.7	13.1	
low-to-moderate drinkers	61.3	60.3	59	60.8	
heavy drinkers	25.4	24.7	27.3	26.1	
Smoking status (%)					<0.001
Never smokers	55.8	57.1	51.7	46.7	
Former smokers	23.2	25.3	23.7	26.3	
Current smokers	21	17.6	24.6	27	
CHD (%)					0.797
Yes	2.4	3.1	2.9	3.3	
No	97.6	96.9	97.1	96.7	
Hypertension (%)					<0.001
Yes	21.3	22	26	29.7	
No	78.7	78	74	70.3	
Total cholesterol, mmol/L	4.665 ± 1.045	4.819 ± 1.135	4.882 ± 1.059	4.898 ± 1.041	<0.001
HDL-C, mmol/L	1.424 ± 0.400	1.400 ± 0.386	1.374 ± 0.364	1.420 ± 0.398	0.013
Triglyceride, mmol/L	1.232 ± 1.243	1.319 ± 0.988	1.376 ± 0.963	1.356 ± 0.875	0.008
LDL-C, mmol/L	2.694 ± 0.845	2.824 ± 0.962	2.888 ± 0.906	2.858 ± 0.903	<0.001
FPG, mmol/L	5.365 ± 0.490	5.403 ± 0.522	5.414 ± 0.516	5.445 ± 0.543	0.005
HbA1c, %	5.298 ± 0.396	5.260 ± 0.372	5.296 ± 0.376	5.334 ± 0.381	<0.001
Two hour OGTT, mmol/L	5.655 ± 1.639	5.887 ± 1.738	6.003 ± 1.707	6.284 ± 1.810	<0.001
HOMA-IR	2.658 ± 2.177	2.760 ± 2.224	2.912 ± 2.432	3.022 ± 2.478	0.002

Mean ± SD for continuous variables: the *p*-value was calculated by a weighted linear regression model. % for categorical variables: the *p*-value was calculated by a weighted chi-square test. Q, quartile; BMI, body mass index; PIR, poverty income ratio; CHD, coronary heart disease; HDL-C, high-density lipoprotein cholesterol; LDH-C, low-density lipoprotein cholesterol; FPG, fasting plasma glucose; HbA1c, hemoglobin A1c; HOMA-IR, homeostatic model assessment of insulin resistance.

### Association between SII and glucose regulation

Table 2 highlights the relationships between SII and markers of glucose regulation. After full adjustment for covariates in Model 3, participants in the highest quartile of SII score showed significantly elevated OGTT levels compared to those in the first quartile of SII score (β coefficients = 0.421, 95% CI: 0.223, 0.619; *p* for trend < 0.001). However, no significant associations were observed between SII and FPG, HbA1c, or FSI.

**Table 2:** β-Coefficients (95%CIs) for the relationship between SII and the markers of glucose metabolism (National Health and Nutrition Examination Survey 2005–2016).

Outcomes	β (95% CI)				*p* for trend
	Q1	Q2	Q3	Q4	
	N = 1031	N = 1030	N = 1030	N = 1031	
FPG, mmol/L					
Model 1	ref	0.038 (-0.007, 0.083)	0.050 (0.005, 0.095)	0.081 (0.036, 0.125)	0.004
Model 2	ref	0.008 (-0.035, 0.051)	0.018 (-0.025, 0.060)	0.031 (-0.012, 0.075)	0.085
Model 3	ref	-0.043 (-0.102, 0.016)	-0.009 (-0.068, 0.051)	-0.004 (-0.063, 0.055)	0.766
HbA1c, %					
Model 1	ref	-0.038 (-0.071, -0.005)	-0.003 (-0.036, 0.030)	0.035 (0.002, 0.068)	<0.001
Model 2	ref	-0.025 (-0.056, 0.005)	0.002 (-0.028, 0.032)	0.014 (-0.017, 0.045)	0.007
Model 3	ref	-0.060 (-0.102, -0.019)	-0.032 (-0.074, 0.009)	-0.005 (-0.046, 0.037)	0.421
OGTT, mmol/L					
Model 1	ref	0.232 (0.083, 0.381)	0.348 (0.199, 0.497)	0.629 (0.481, 0.778)	<0.001
Model 2	ref	0.145 (0.002, 0.289)	0.220 (0.077, 0.364)	0.365 (0.219, 0.511)	<0.001
Model 3	ref	0.099 (-0.101, 0.298)	0.199 (-0.001, 0.399)	0.421 (0.223, 0.619)	<0.001
FSI, μU/ml					
Model 1	ref	-0.588 (-1.377, 0.201)	0.041 (-0.740, 0.822)	0.613 (-0.166, 1.393)	0.018
Model 2	ref	-0.242 (-1.032, 0.548)	0.356 (-0.427, 1.139)	1.183 (0.391, 1.974)	<0.001
Model 3	ref	-0.693 (-1.336, -0.051)	-0.761 (-1.402, -0.121)	-0.091 (-0.743, 0.560)	0.967

Model 1 adjust for: None.

Model 2 adjust for: gender, age, race, PIR.

Model 3 adjust for: gender, age, race, PIR, BMI, drinking status, smoking status, CHD, hypertension, total cholesterol, triglyceride, HDL-C and LDL-C level

FPG, fasting plasma glucose; HbA1c, glycohemoglobin; OGTT, oral glucose tolerance test; FSI, fasting serum insulin.

**Fig 2:**
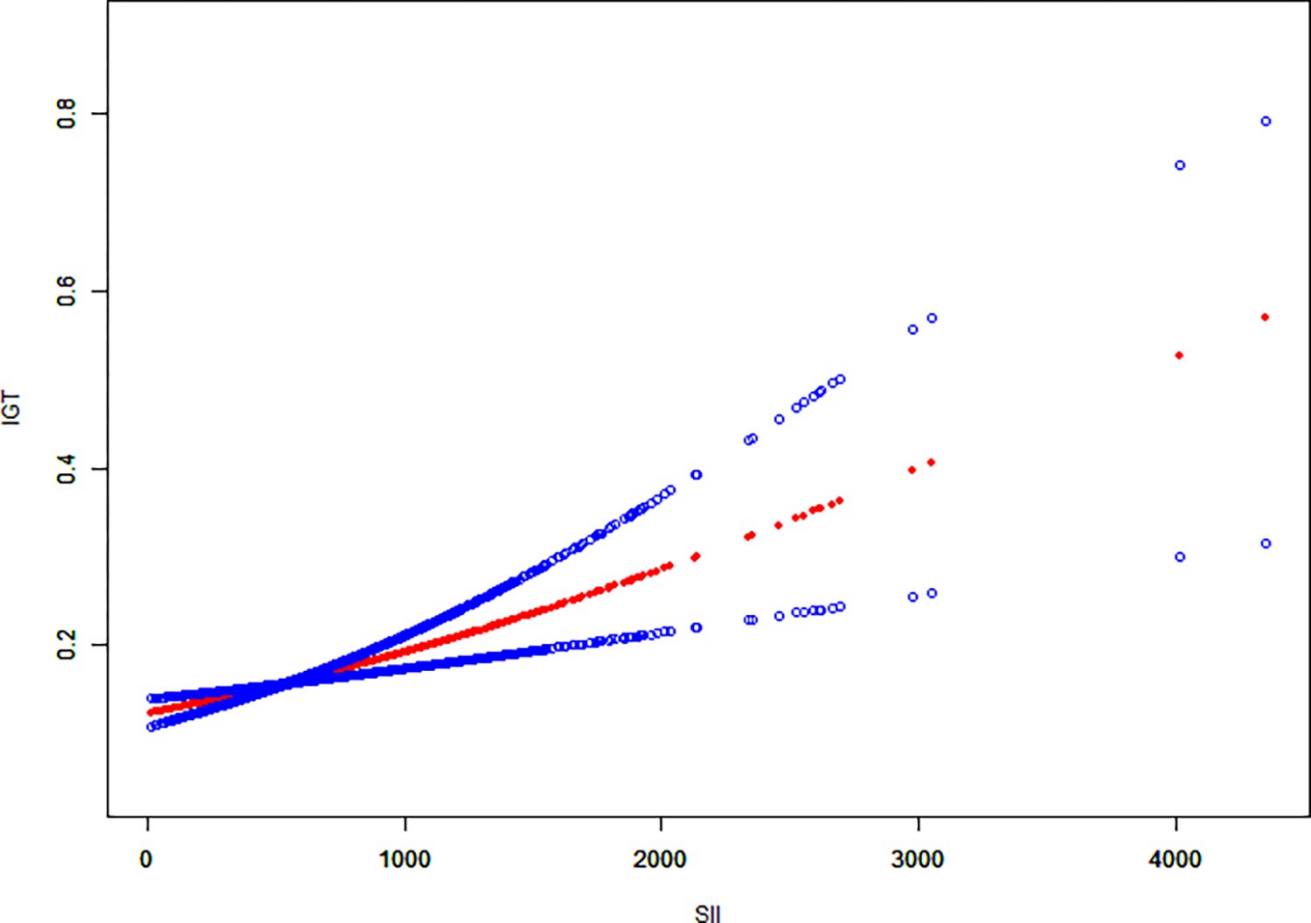
Smooth curve fitting for SII and IGT.

The association between SII and glucose regulation status based on SII quartiles is presented in Table 3. Initially, when no covariates were adjusted (model 1) and when adjusted for gender, age, race, and PIR (model 2), a higher SII score was linked to an increased risk of insulin resistance (IR). However, when additional covariates were included in the analysis (model 3), the positive association between SII and IR became insignificant. Notably, a higher SII score was associated with an increased risk of prediabetes only in model 1. Due to the significantly higher OGTT levels in participants in the highest SII quartile compared to those in the lowest quartile, an association between SII and impaired glucose tolerance (IGT) was observed. The multivariable-adjusted odds ratios (95% CIs) for IGT in the second, third, and fourth quartiles of SII in model 3 were 1.555 (95% CI: 1.048, 2.307), 1.759 (95% CI: 1.194, 2.592), and 2.673 (95%CI: 1.845, 3.873), respectively (*p* for trend < 0.001). Participants in the fourth quartile exhibited a 167% increased risk of IGT compared to those in the lowest quartile. In addition, the results of smooth curve fitting indicated that there was no non-linear relationship between SII and the risk of IGT (Fig 2).

**Table 3:** Multivariable-adjusted odds ratios (95%CIs) of glucose metabolism status according to the quartiles of SII (National Health and Nutrition Examination Survey 2005–2016).

Outcomes	OR (95% CI)				*p* for trend
	Q1	Q2	Q3	Q4	
	N = 1031	N = 1030	N = 1030	N = 1031	
Prediabetes					
Model 1	ref	0.970 (0.815, 1.155)	1.167 (0.981, 1.389)	1.309 (1.101, 1.557)	<0.001
Model 2	ref	0.916 (0.750, 1.118)	1.122 (0.920, 1.369)	1.118 (0.913, 1.368)	<0.001
Model 3	ref	0.833 (0.631, 1.100)	1.125 (0.852, 1.486)	1.053 (0.799, 1.387)	<0.001
IR					
Model 1	ref	1.249 (1.013, 1.540)	1.493 (1.217, 1.833)	1.603 (1.308, 1.965)	<0.001
Model 2	ref	1.306 (1.055, 1.616)	1.538 (1.248, 1.897)	1.718 (1.391, 2.122)	<0.001
Model 3	ref	1.053 (0.816, 1.360)	1.039 (0.805, 1.341)	1.144 (0.881, 1.485)	0.354
IGT					
Model 1	ref	1.453 (1.117, 1.890)	1.660 (1.283, 2.149)	2.335 (1.822, 2.991)	<0.001
Model 2	ref	1.320 (0.987, 1.765)	1.492 (1.123, 1.984)	1.839 (1.395, 2.424)	<0.001
Model 3	ref	1.555 (1.048, 2.307)	1.759 (1.194, 2.592)	2.673 (1.845, 3.873)	<0.001

Model 1 adjust for: None.

Model 2 adjust for: gender, age, race, PIR.

Model 3 adjust for: gender, age, race, PIR, BMI, drinking status, smoking status, CHD, hypertension, total cholesterol, triglyceride, HDL-C and LDL-C level

IR, insulin resistance; IGT, impaired glucose tolerance.

**Fig 3:**
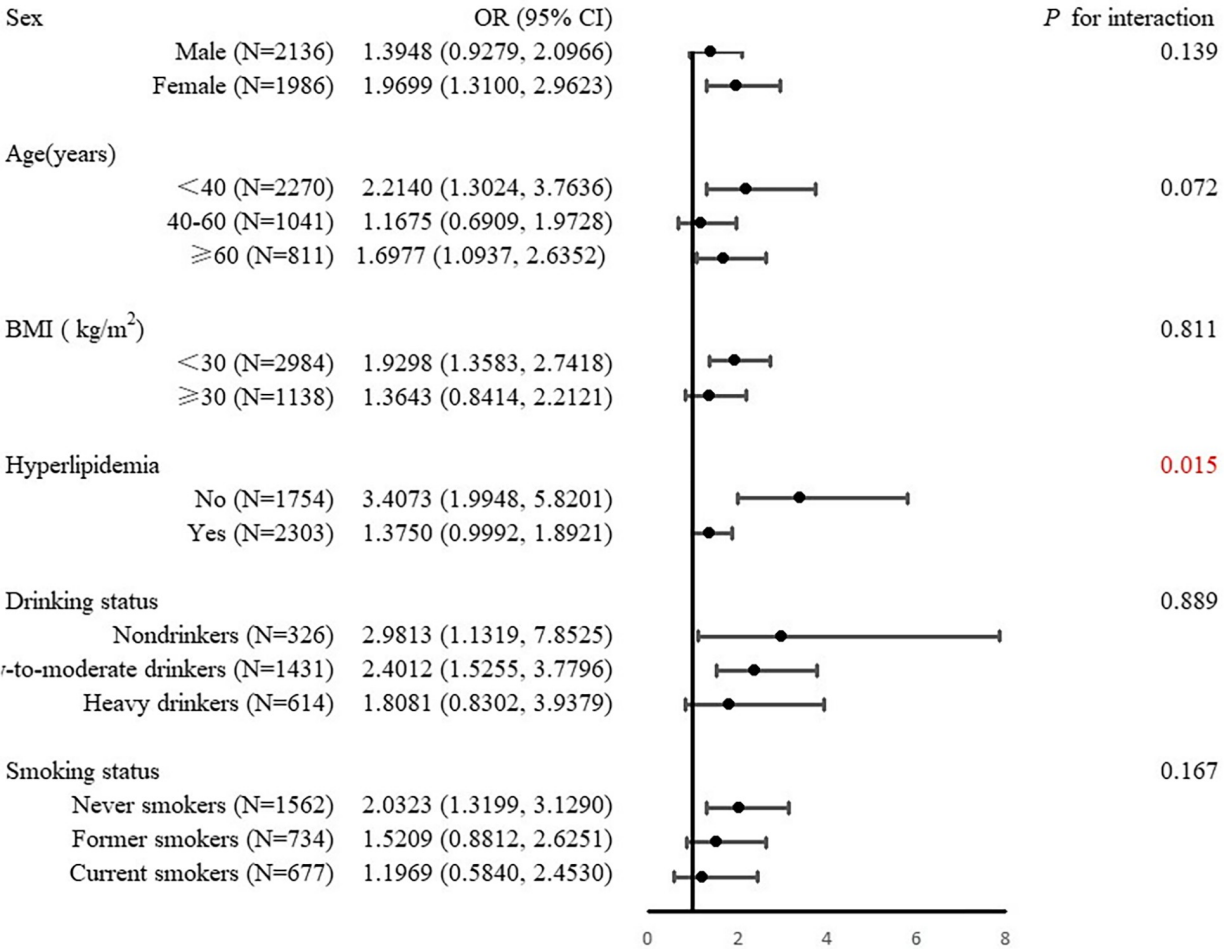
Subgroup analysis for the association between systemic immunity-inflammation index and impaired glucose tolerance.

Subgroup analysis revealed inconsistencies in the relationship between SII and IGT (Fig 3). After adjusting for confounding factors, we found that participants without hyperlipidemia in the highest SII quartile had a 240% higher odds of IGT than those in the lowest quartile (OR = 3.407, 95%CI: 1.995, 5.820). This risk was not observed in participants with hyperlipidemia (OR = 1.375, 95%CI: 0.999, 1.892) (*p* for interaction < 0.05). We also found that females, age below 40 and above 60, non-obese (BMI < 30 kg/m^2^), nondrinkers, low-to-moderate drinkers, and never smokers in the highest quartiles of SII had a higher odds of IGT than those in the lowest quartile. However, interaction tests revealed that age, gender, BM, drinking status, and smoking status did not significantly impact this positive correlation (*p* for interaction > 0.05). Subgroup analyses stratified by gender, age, BMI, hyperlipidemia, drinking status, and smoking habits did not reveal any significant association between SII and prediabetes or IR (see S1 and S2 Tables).

## Discussion

To our knowledge, this study is the first to establish a significant association between SII and abnormal glucose regulation in the non-diabetic population. Our results reveal a positive correlation between SII levels and OGTT levels. Moreover, we have identified that higher SII levels are independently linked to IGT in non-diabetic individuals, especially those without hyperlipidemia.

The global prevalence of IGT was 9.1% (464 million) in 2021 and is projected to increase to 10.0% (638 million) by 2045 [23]. IGT is recognized to precede T2D and can be attributed to IR, decreased insulin secretion, or a combination of both factors. Pro-inflammatory cytokines have been shown to negatively impact insulin sensitivity and β-cell function, potentially leading to IR development and T2D pathogenesis [10, 24]. Elevated levels of the pro-inflammatory marker C-reactive protein (CRP) and pro-inflammatory cytokines have been observed in both IGT and T2D, predicting the progression to T2D [25–31]. This evidence supports the idea that IGT involves a subclinical pro-inflammatory state. However, there is no widely accepted inflammatory marker that can consistently predict the onset of IGT. While CRP appears to have a stronger and more consistent association with the increased risk of developing T2D [32], a previous study by Krakoff et al. [33] had not found CRP levels to be predictive of diabetes. In a study focusing on elderly Korean women, levels of thetokine interleukin 6 (IL-6) were unchanged in those with IGT [34], contrasting with other research showing higher IL-6 concentrations in individuals with IGT [25].

The SII is increasingly recognized as a reliable biomarker for assessing overall immune system activity and inflammatory status [12–15]. Previous studies have linked higher SII levels to an increased incidence of diabetes [35], diabetic macular edema [36], diabetic depression [37], and diabetic kidney disease [38]. Based on these findings in diabetic populations, we hypothesized a potential correlation between SII and abnormal glucose regulation in non-diabetic individuals, suggesting that SII could serve as a promising predictive marker. Our study, focusing on non-diabetic individuals, found that elevated SII levels are independently associated with a higher risk of IGT, particularly in those without hyperlipidemia. These results support the role of SII in the development of glucose regulation abnormalities and underscore the importance of monitoring SII levels in non-diabetic individuals without hyperlipidemia, who may face an elevated risk of developing abnormal glucose regulation conditions. Further research is warranted to understand the underlying mechanisms behind these observations.

## Conclusions

In conclusion, the SII has the potential to serve as a diagnostic biomarker for identifying IGT in non-diabetic individuals, especially those without hyperlipidemia. Its inclusion in risk stratification and management strategies could improve the detection and individualized treatment of abnormal glucose regulation, ultimately enhancing outcomes in the non-diabetic population.
